# Focused x-ray luminescence imaging system for small animals based on a rotary gantry

**DOI:** 10.1117/1.JBO.26.3.036004

**Published:** 2021-03-18

**Authors:** Michael C. Lun, Wenxiang Cong, Mohammad Arifuzzaman, Meenakshi Ranasinghe, Sriparna Bhattacharya, Jeffrey N. Anker, Ge Wang, Changqing Li

**Affiliations:** a University of California, Merced, Department of Bioengineering, Merced, California, United States; b Rensselaer Polytechnic Institute, Biomedical Imaging Center, Center for Biotechnology and Interdisciplinary Studies, Department of Biomedical Engineering, Troy, New York, United States; c Clemson University, Department of Chemistry, Clemson, South Carolina, United States; d Clemson University, Clemson Nanomaterials Institute, Department of Physics and Astronomy, Clemson, South Carolina, United States; e Clemson University, Institute of Environmental Toxicology, Center for Optical Materials Science and Engineering Technology, Department of Bioengineering, Clemson, South Carolina, United States

**Keywords:** x-ray imaging, optical tomography, molecular imaging, deep learning, nanoparticles

## Abstract

**Significance:** The ability to detect and localize specific molecules through tissue is important for elucidating the molecular basis of disease and treatment. Unfortunately, most current molecular imaging tools in tissue either lack high spatial resolution (e.g., diffuse optical fluorescence tomography or positron emission tomography) or lack molecular sensitivity (e.g., micro-computed tomography, μCT). X-ray luminescence imaging emerged about 10 years ago to address this issue by combining the molecular sensitivity of optical probes with the high spatial resolution of x-ray imaging through tissue. In particular, x-ray luminescence computed tomography (XLCT) has been demonstrated as a powerful technique for the high-resolution imaging of deeply embedded contrast agents in three dimensions (3D) for small-animal imaging.

**Aim:** To facilitate the translation of XLCT for small-animal imaging, we have designed and built a small-animal dedicated focused x-ray luminescence tomography (FXLT) scanner with a μCT scanner, synthesized bright and biocompatible nanophosphors as contrast agents, and have developed a deep-learning-based reconstruction algorithm.

**Approach:** The proposed FXLT imaging system was designed using computer-aided design software and built according to specifications. ${\mathrm{NaGdF}}_{4}$ nanophosphors doped with europium or terbium were synthesized with a silica shell for increased biocompatibility and functionalized with biotin. A deep-learning-based XLCT image reconstruction was also developed based on the residual neural network as a data synthesis method of projection views from few-view data to enhance the reconstructed image quality.

**Results:** We have built the FXLT scanner for small-animal imaging based on a rotational gantry. With all major imaging components mounted, the motor controlling the gantry can be used to rotate the system with a high accuracy. The synthesized nanophosphors displayed distinct x-ray luminescence emission, which enables multi-color imaging, and has successfully been bound to streptavidin-coated substrates. Lastly, numerical simulations using the proposed deep-learning-based reconstruction algorithm has demonstrated a clear enhancement in the reconstructed image quality.

**Conclusions:** The designed FXLT scanner, synthesized nanophosphors, and deep-learning-based reconstruction algorithm show great potential for the high-resolution molecular imaging of small animals.

## 1 Introduction

During the past decade, the concept of x-ray luminescence imaging (XLI) emerged and demonstrated great potential for molecular imaging in small animals by combining the high-spatial resolution of traditional x-ray imaging and the superb measurement sensitivity of optical imaging. Specifically, x-ray luminescence computed tomography (XLCT) is a powerful imaging modality capable of the high-resolution imaging of deeply embedded x-ray excitable contrast agents in 3D. In principle, a focused or collimated beam of x-ray photons are used to penetrate deeply through the tissue samples, with minimal scatter; x-ray excitable contrast agents within the x-ray beam path absorb the x-ray energy, causing a photophysical cascade of events, eventually leading to the emission of many optical photons, which can then pass through the tissue and escape from the skin. Highly sensitive photodetectors, such as electron multiplying charge-coupled device (EMCCD) cameras or photomultiplier tubes (PMTs), can then detect the optical photons for image reconstruction. Pratx et al.^1^^,^^2^ first reported a selective-excitation-based XLCT imaging and demonstrated that the distribution of deeply embedded contrast agents could be recovered with high-resolution and sensitivity with this method. Soon following, several research groups, including our own, improved several aspects of XLCT including the XLCT imaging systems,^3^^–^^13^ robust reconstruction algorithms,^4^^,^^14^ and designing efficient, bright, and biocompatible XLCT imaging probes.^15^^–^^20^

XLCT can be performed using different x-ray beam geometries, each with their own advantages and disadvantages. Selective beam-based XLCT is performed using a narrow x-ray beam that raster scans across the sample such as the first-generation computed tomography (CT) scanners. This allows for very high-spatial resolution since the beam size and location can be used as *a priori* information in the image reconstruction. The drawback of this method is the inherently long scan time due to the small excitation region and time needed to traverse the whole object. Another geometry is the fan or planar beam geometry, which can be used to image an entire cross-section at one time and allows for selective-planar imaging.^11^^,^^12^ This method eliminates the need for raster scanning, which improves the imaging time; however, the lateral spatial resolution is degraded compared with the narrow beam selective excitation since the excitation is no longer localized to a small region but rather the entire cross-section. The last x-ray beam geometry is the conical-beam geometry, which offers the fastest scanning time of the three geometries mentioned since the object is entirely excited by the beam at one time.^13^ However, due to the *ill-posedness* of the image reconstruction, this method further compromises the spatial resolution as a trade-off. Due to advantages of the improved spatial resolution, our efforts have focused on the narrow x-ray beam-based XLCT imaging.

We performed narrow x-ray beam-based XLCT imaging using collimators to generate a fine x-ray beam and used an EMCCD camera to detect the optical photons that reach the object surface.^3^^–^^6^^,^^21^ With this method, we achieved promising results and demonstrated that we could separate targets with an edge-to-edge distance of 0.15 mm^22^ and could perform XLCT imaging at depths greater than 2 cm and at concentrations of 0.01  mg/ml (${\mathrm{Gd}}_{2}O_{2}S:{\mathrm{Eu}}^{3+}$ contrast agent).^21^ To improve on reducing the imaging time, a challenge for the narrow-beam-based XLCT, we also proposed the idea of using a multiple-pinhole collimator to generate multiple scanning x-ray beams and thereby reduced the imaging time by a factor equal to the number of beams.^6^ More recently, we have explored using both a higher-flux x-ray beam and more sensitive optical detectors to perform XLCT imaging. Collimation, while easy to implement, is highly inefficient as most photons generated by the x-ray source are absorbed by the collimator, with only a small number of photons able to pass through the pinhole. We recently demonstrated that we can increase the x-ray photon flux, and thus perform much more sensitive XLCT using x-ray optics (e.g., a polycapillary lens), to capture divergent x-ray photons from the source and refocus them with a dual-cone geometry. Also, using PMTs instead of an EMCCD camera, we can acquire our optical signal more rapidly and with a higher signal-to-background ratio.^7^

XLCT relies on x-ray luminescence scintillators to generate light in the tissue with minimal background from the endogenous tissue. Rare earth-doped nanophosphors are promising XLCT contrast agents for noninvasive bioimaging, such as to detect tumors *in vivo*. For such applications, the nanophosphors should ideally be small, colloidally stable, and chemically functionalizable for targeting, and generate bright radioluminescence signals with tissue penetrating red or near-infrared (NIR) light. Many microphosphors are commercially available, mostly developed for lighting, display, and x-ray imaging applications. Smaller stable nanophosphors have also been developed for biolabeling applications, especially fluorescence and upconversion, and some of these (e.g., ${\mathrm{NaGdF}}_{4}:\mathrm{Eu}$ and ${\mathrm{Gd}}_{2}O_{2}S:\mathrm{Eu}$) are also good XLI contrast agents.^2^^,^^15^^–^^20^ Reducing the phosphor size usually decreases their luminescence yield due to quenching from surface defects and escape of x-ray generated photoelectrons, but annealing processes can help by reducing quenching from defects. Based on our previous work with XLCT imaging, along with the efforts of our collaborators, we have proposed and are currently building a small-animal dedicated focused x-ray luminescence tomography (FXLT) imaging system that also incorporates a micro-computed tomography (μCT) scanner. We also synthesized nanophosphors with various emission wavelengths to be used as contrast agents for multiplexed XLCT imaging. Lastly, we propose a deep-learning-based XLCT reconstruction algorithm to improve upon our existing reconstruction methods. The remainder of the paper is arranged as follows. In Sec. 2, we discuss the design and build of our designed FXLT imaging system and the proposed scanning scheme. In Sec. 3, we discuss the proposed synthesized nanophosphors. In Sec. 4, we discuss the deep-learning-based XLCT reconstruction algorithm and numerical simulations. Lastly, Sec. 5 presents the summary and conclusion.

## 2 FXLT Imaging System Design and Scanning Scheme

**Fig. 1:**
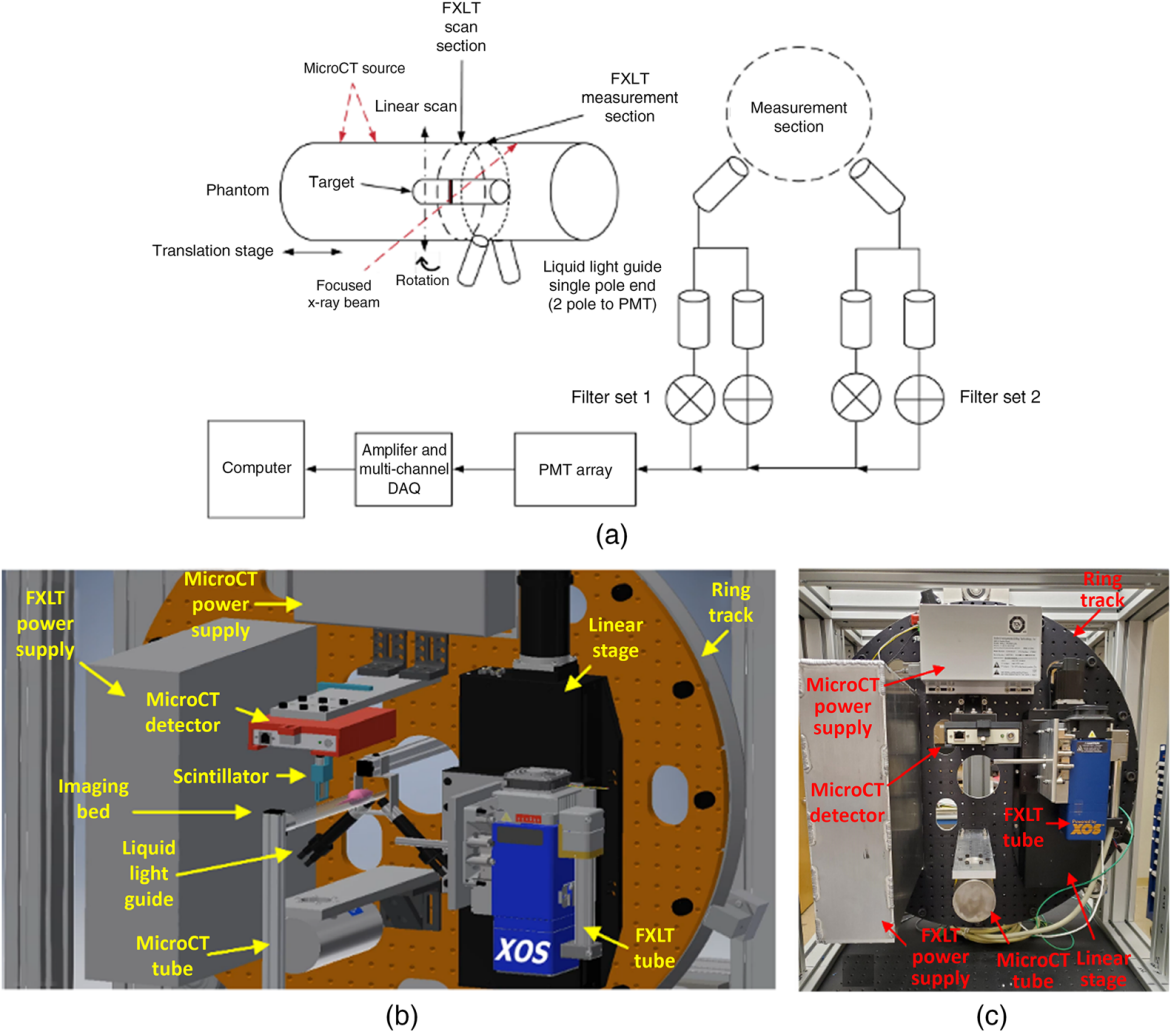
The FXLT Imaging System. (a) Proposed scanning scheme for the FXLT system; (b) CAD model of the proposed system; and (c) physical build of the scanner.

The proposed FXLT scanner was designed by computer-aided design (CAD) software and built at our Biomedical Imaging Lab at the University of California, Merced, as shown in Figs. 1(b) and 1(c). The imaging system frame was constructed from extruded T-slotted aluminum bars (80/20 Inc.) and has approximate dimensions of 2.82  ft.×3.90  ft.×5.77  ft. (width×length×height). Custom lead-lined stainless-steel panels and door were designed and fabricated (BFK Innovation Inc.) and were mounted to the frame to protect from x-ray leakage as well as allowing for the system to be light-tight.

### 2.1 Rotary Gantry

A custom cut optics board was mounted onto a heavy-duty ring track (HDRT) (Hepco Motion), which was mounted to the imaging system frame. The HDRT has a central bore diameter of 650 mm and contains an internal V-track that uses a pinion and shaft system to rotate for different angular projections. We used a powerful high-precision servomotor (SGM7A-04A6A6C, Yaskawa) with an attached gearhead (ValueTRUE 6, Boston Gear) to drive the pinion. The servomotor has an output power of 400 watts (W) and runs from a power supply using 200 VAC with a torque rating of 1.27  N/m. Included with the servomotor is a 24-bit batteryless absolute encoder to track the absolute position of the motor and a holding brake to stop unnecessary movements of the gantry. The additional gearhead is used to provide sufficient torque to drive the load from the rotary gantry and has a gear ratio of 7:1. From the perspective of the motor and gearhead, an accuracy of 0.067 deg can be achieved. To fix the motor to the gantry, we first used a rigid coupler to attach a stainless-steel pinion shaft (30 mm diameter, h8 tolerance) to the gearhead shaft. Then we coupled the pinion shaft to the pinion (HP4X24, Hepco Motion), which contains a keyless locking bush for securing the shaft. The motor was then fixed to a custom L-shaped bracket and mounted onto the imaging system frame from behind the gantry and aligned with the internal V-track to rotate the gantry. All the primary imaging system components were fixed onto the optics board. These components include two x-ray tubes (one for performing μCT imaging and the other for FXLT), their associated power supplies, an x-ray detector, and a heavy-duty linear stage for translating the FXLT x-ray tube.

### 2.2 Scan Scheme of the FXLT Scanner

Figure 1(a) summarizes the scanning scheme of the FXLT scanner. As seen in Fig. 1(b), a linear stage is used to position and bring our imaged object into the field-of-view (FOV) of the scanner components that are mounted to the HDRT. First, the object enters the FOV of the μCT scanner. For the μCT scanner, we have an x-ray tube (XTF5011, Oxford Instruments) fixed to an aluminum bracket. Opposite from the x-ray tube, we mounted an x-ray detector (Shad-o-Box 1K HS, Teledyne DALSA) to collect our μCT projection images. We also fixed the power supply (Shasta Power Supply) to the ring track as well. Following the μCT scan, the object is then positioned into the FOV of the FXLT scanner to perform x-ray optical imaging. Here, we used a powerful focused x-ray tube with polycapillary lens (fleX-Beam, XOS) that has an approximate focal spot size of 49.9  μm [FWHM, $\mathrm{Mo}\text{-}K_{\alpha }$ (17.4 keV)] and an x-ray photon flux of $2.8\times 10^{7}\text{ photons}/s$ (at 50 keV). The x-ray beam convergence angle is less than 2 deg and the beam diameter changes to a maximum of 75  μm within 10 mm from the focal spot. The power supply (PCS-50) is also fixed and mounted to the ring track using a custom-designed aluminum box. The focused x-ray tube is fixed onto a heavy-duty precision linear stage (NLS-8, Newmark Systems Inc.) that allows for translational scanning of the focused x-ray beam. Directly opposite of the polycapillary lens, we fixed a small scintillator crystal (e.g., $\mathrm{Ce}:{\mathrm{Lu}}_{2}{\mathrm{SiO}}_{5}$) that can constantly track the intensity of the focused beam by delivering the signal to a PMT through an optical fiber bundle. This is used to determine when an object (e.g., mouse or phantom) is in the path of the x-ray beam for boundary determination during FXLT image reconstruction.

### 2.3 FXLT Measurements

During the FXLT scans, emitted optical photons that reach the object surface are delivered to an array of four PMTs (H7422-50, Hamamatsu Inc.) by use of two bifurcated liquid-light guides (Series 380, Lumatec) that are optimized for the NIR range (up to 800 nm) with high efficiency. During the FXLT scans, the light guides are fixed and do not rotate and are positioned a few millimeters from the FXLT scan section at the FXLT measurement section as shown in Fig. 1(a), so the x-ray beam does not impinge on the fiber entrances during scanning. Before the optical signal is delivered to the PMTs, we apply a bandpass filter to select two (or up to a maximum of four) wavelengths of interest, which allows for us to perform multiplexed (multicolor) imaging. Then the PMT output signal is further amplified by use of a preamplifier (SR445A, Stanford Research Systems) and then a low-pass filter is applied (BLP-10.7+, Cutoff Frequency: 11 MHz, Mini-Circuits) to reduce high-frequency noises before the signal is finally collected by a high-speed digitizer (DTS5730S, CAEN Technologies Inc.) connected to a lab computer.

## 3 X-ray Excitable Contrast Agents

**Fig. 2:**
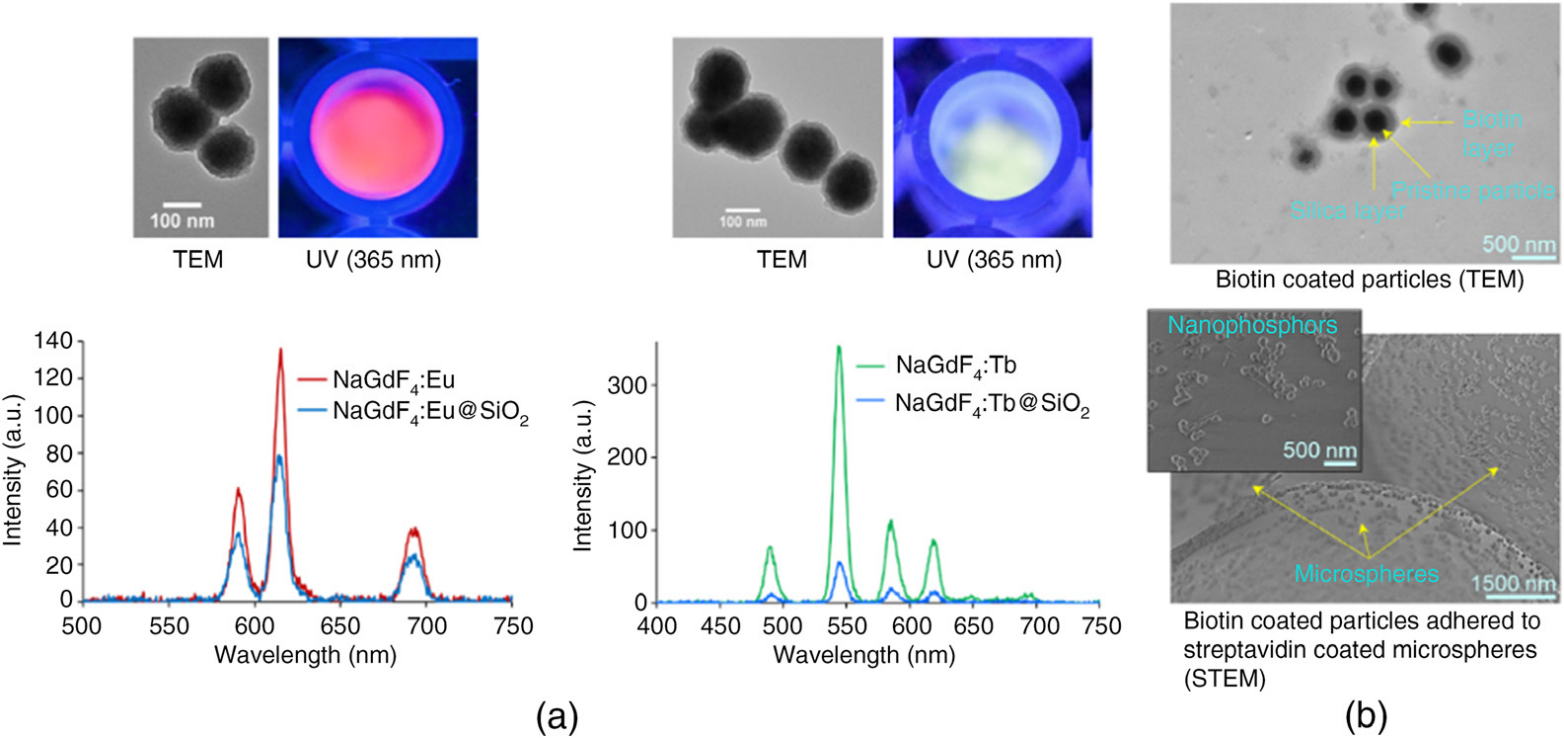
(a) Luminescence spectra of ${\mathrm{NaGdF}}_{4}:\mathrm{Eu}$ (red) and ${\mathrm{NaGdF}}_{4}:\mathrm{Tb}$ (green) nanophosphors, TEM images above the spectra show silica coated particles. (b) TEM image of biotin-coated nanophosphors (top), and STEM image of biotin-coated nanophosphors adhered to streptavidin coated microspheres (bottom), inset image is a zoomed-in view.

We will employ rare earth-doped nanophosphors as contrast agents in this multiplexed bioimaging system. For example, ${\mathrm{NaGdF}}_{4}:\mathrm{Eu}$ or Tb was synthesized using a citrate method, involving lanthanides-citrate complex formation followed by nucleation and growth upon the addition of NaF. Synthesized nanoparticles were washed and finally dispersed in aqueous solution (pH 7). Following the synthesis, the nanoparticles were annealed at a high temperature (above 850°C) to increase the emission intensity by removing defects that can cause luminescence quenching. To prevent particles sintering during this annealing, ${\mathrm{NaGdF}}_{4}$ nanoparticles were encapsulated within a silica shell using the Stöber process, as described below. The synthesized nanoparticles were characterized using dynamic light scattering (DLS), transmission electron microscopy (TEM), and x-ray excited optical luminescence (XEOL) spectroscopy (Fig. 2).

Luminescent ${\mathrm{NaGdF}}_{4}$ nanoparticles were encapsulated with silica layers via the Stöber process, modified with (3-aminopropyl) triethoxysilane, and lastly functionalized with carboxylic acid–PEG–Biotin (1000 Da) using EDC coupling chemistry in aqueous MES buffer. The surface functionalization with biotin was initially verified by scanning electron microscopy imaging, which showed that biotinylated nanophosphors adhered to streptavidin-coated silica microspheres. Surface charge and size of the biotinylated nanophosphors were measured using DLS.

Synthesized nanoparticles emit visible light photons when irradiated with x-rays. Both DLS measurement and TEM indicate that the size of the nanophosphors without any modifications is around 100 nm. According to XEOL spectroscopy measurements, both Eu- and Tb-doped ${\mathrm{NaGdF}}_{4}$ nanophosphors show relatively low emission intensity, likely due to lack of luminescent centers or self-quenching, respectively. Later, hydrothermal treatment increased the emission intensity by a factor of 2 to 3 (annealing without a silica shell increased the emission intensity by another factor of 5 but resulted in sintered particles, negating their biological application). After annealing silica-coated ${\mathrm{NaGdF}}_{4}$ particles, there was minimal increase in intensity compared to pristine nanoparticles. Later, silica shelled nanophosphors were functionalized with biotin-labeled PEG molecules. Post functionalization steps, the nanophosphors bind well with a streptavidin-coated glass beads (Akadeum Life Sciences) demonstrating functionalization. Additional information and comparisons using the nanophosphors can be found in Ref. 23.

## 4 Machine Learning-Based XLCT Reconstruction Algorithm

### 4.1 X-ray Luminescence Imaging Model

The propagation of luminescence light through the biological tissue is a complex process, and experiences both absorption and scattering simultaneously. A light propagation model describes the interaction of photons with scattering and absorbing media and is essential for optical tomographic imaging. The radiative transfer equation is an accurate model for photon propagation in biological tissues and can be solved with a numerical solution or Monte Carlo simulation. From photon propagation model, we can express photon fluence rates on an object surface in terms of a Green function:^24^^,^^25^

$$\Phi (r)=∭G(r,r^{\prime })S(r^{\prime })dr^{\prime },\;r\in \partial \Omega \;,$$ (1)

where Ω is the imaging region of interest, r is a positional vector, Φ(r) is the photon fluence rate at a location r ($W/{\mathrm{mm}}^{2}$), and S(r) the intensity of the luminescent light source ($W/{\mathrm{mm}}^{3}$) in the object. The intensity of luminescence emitted from nanophosphors is related to the nanophosphor concentration ρ(r) to be reconstructed, the x-ray intensity distribution, X(r) in the imaging object, which is determined by the Beer–Lambert law, and the luminescence yield η of the nanophosphors, which is defined as the quantum yield per unit nanophosphor ${\text{concentration}}^{2}$: S(r)=ηX(r)ρ(r).

Equation (1) is a linear integral equation with respect to nanophosphors concentration, which can be discretized into a matrix equation linking the nanophosphor distribution ρ(r) and the photon fluence rate Φ(r) at measurable position r,^14^^,^^26^

Φ=A·ρ, (2)

where A is the weight matrix of nanophosphor distribution. Equation (2) describes a linear relationship between the nanophosphor concentration and measured data. In the x-ray luminescence imaging, a focused x-ray beam irradiates the object to excite nanophosphors. The nanophosphors on the x-ray transmission path are the potential luminescent light source S(r) only. The x-ray luminescence imaging is very similar to the first-generation CT imaging modality and adopts a parallel x-ray beam imaging mode for the luminescence imaging at a projection view. Then, the direction of the x-ray beam is rotated to acquire images at a different projection view. Thus, we can acquire projection data at multiple views for the image reconstruction of nanophosphors concentration. The image reconstruction is to localize and quantify a nanophosphors concentration distribution in a small animal based on measured luminescence photon fluence rates. Using projection data, the image reconstruction can be performed based on Eq. (2) using an iterative algorithm combined with regularization techniques.

### 4.2 Synthesis of Projection Data

The x-ray luminescence imaging adopts the first-generation CT imaging modality and would take a lot of scanning time. An important method to enhance XLCT scanning efficiency is to reduce the number of projection views and x-ray beam translations. Here, we develop a data-driven interpolation method of projection views from measured few-view sinogram using deep learning techniques to significantly enhance image quality of image reconstruction. Deep learning techniques can extract non-linear relationships in a data-driven manner, discovering complicated features and representations, and have offered dramatically improved performance in a variety of reconstruction problems. Here, we use the residual neural network (ResNet) to model the non-linear mapping. ResNet has a great ability to extract complex and detailed features from data more effectively than a generic CNN network. With the introduction of shortcuts, ResNet alleviates overfitting, suppresses vanishing/diverging gradients, and allows for the neural network to converge rapidly.

Our residual network consists of one convolution layer with 64 filters of 3×3 kernels, followed by four residual blocks, two convolution layers with 64 filters of 3×3 kernels, one convolution layer with 32 filters of 3×3 kernels, and the last layer generates only one feature map with a single 3×3 filter as the output. Each residual block works in a feed-forward fashion with the shortcut connection skipping three layers to implement an identity mapping. Each layer is followed by an ReLU activation function. The loss function is formulated as a weighted linear combination of the $l_{1}$ norm and the structural similarity (SSIM) index, which is $\text{Loss}=\alpha \cdot \mathrm{SSIM}+(1-\alpha )\cdot l_{1}$ with an empirical weighting factor α. The network is trained using paired few-view projection data and full-view projection data. Through data training, ResNet generates a nonlinear mapping from measured projection data to a new projection data with minimization of the cost function. The network model under particular kernels and weights is calculated through forward propagation on a training dataset, and learnable parameters are updated according to the loss function through an optimization algorithm called backpropagation, which uses the chain rule to speed up the computation of the gradient for the gradient descent algorithm.

### 4.3 Training Dataset

Numerical simulations are cost-effective in generating training datasets for the supervised learning. The phantom or mouse models constructed by finite element meshes are used to simulate mouse geometry and optical properties. With the circulating blood, nanoparticles would accumulate in the liver, spleen, and tumors. In contrast to small molecule contrast agents that wash out of the tumor quickly, the nanoparticles are stably internalized within organs and tumors. Generally, nanophosphors distribution has a spherical morphology with a narrow size distribution. Lesion or tumor is the target area of luminescent imaging, which is contrast sensitivity. To generate training data, targets are distributed randomly within main organs, and the optical parameters were randomly changed in a biologically relevant range.

For the luminescent imaging, the x-ray source voltage and current were set to 40 keV and 30 mA, respectively, and the x-ray beam was filtered with 0.5 mm Al to filter out low-energy photons. X-ray transmission is simulated by Beer–Lambert law and x-ray luminescence is simulated by Monte-Carlo simulation. The physical models well match experimental data. The phantoms or mouse models are irradiated by x-rays to excite nanophosphors to emit luminescent light, and three optical fiber bundles equiangular distributed upper surface of the phantom to collect the luminescence fluence rate. Based on our proposed x-ray luminescent imaging, the projection data were formed from the luminescence measurements at an angular view. The few-view projection data are generated with an angular step size of 12 deg for 15 angular projections over 180 deg. Correspondingly, the full-view projection data are generated with an angular step size of 6 deg for 30 angular projections over 180 deg. We established 400 phantoms and mouse models with various dimensions, perturbation optical parameters, and random positions and various sizes of targets to generate training dataset. Based on the training dataset, the ResNet is trained to generate a ResNet model to map a few-view sinogram to a full-view sinogram.

### 4.4 Validation of Deep-learning Method

**Fig. 3:**
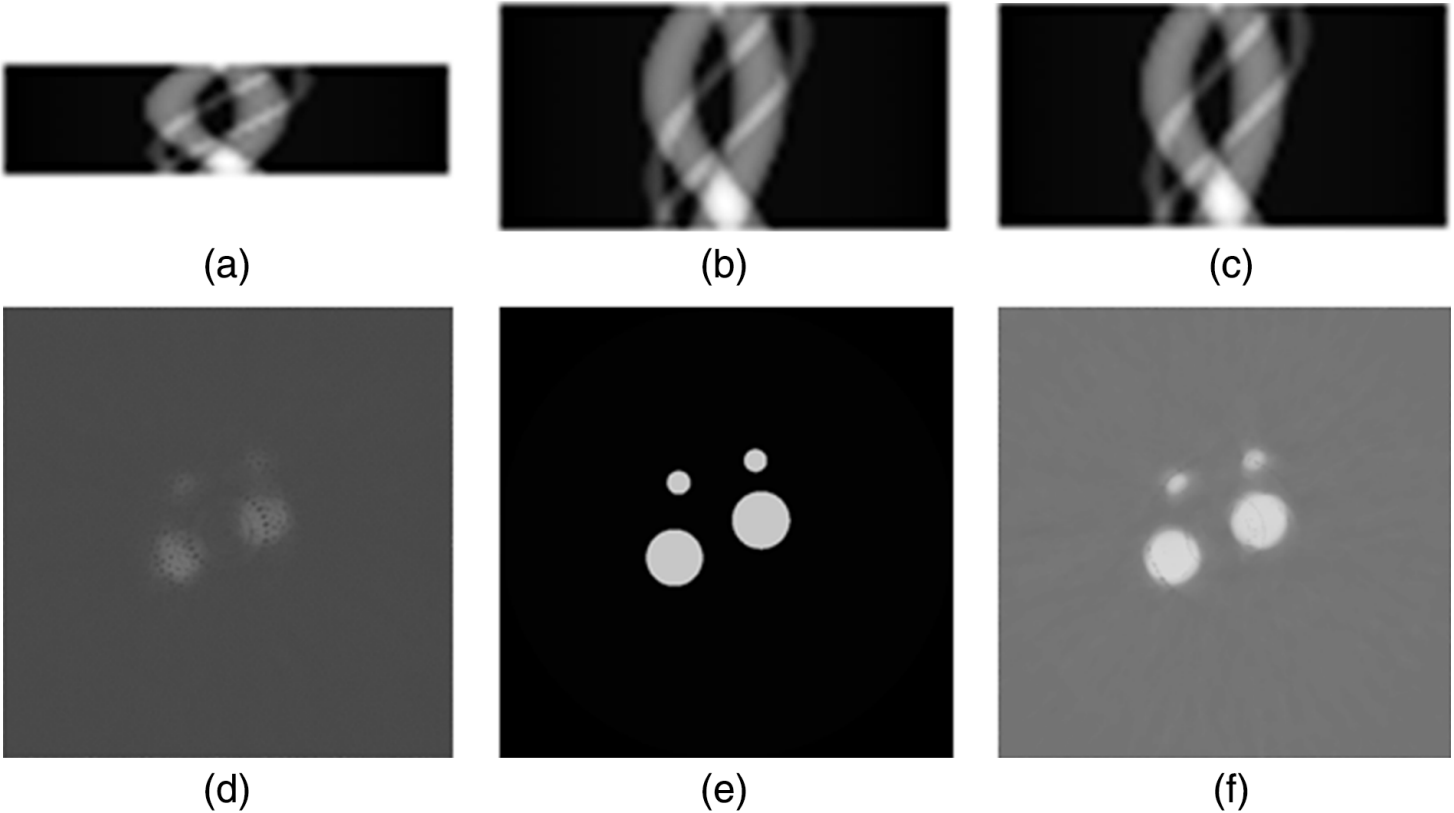
Projection data synthesis and image reconstruction. (a) Sinogram of 15 projection views; (b) sinogram of 30 projection views; (c) reconstructed sinogram from 15 projection views; (d) image reconstructed from 15 projection views; (e) ground truth image of phantom at a plane; and (f) image reconstructed from reconstructed 30 projection views.

We performed testing to demonstrate the proposed deep learning-based image reconstruction method based on 3D digital cylindrical phantoms. The optical parameters of the cylindrical phantoms were set to around $15\;{\mathrm{cm}}^{-1}$ for the scattering coefficient and around $0.5\;{\mathrm{cm}}^{-1}$ for the absorption coefficient. Four target regions were implanted in the phantom. The phantoms were irradiated by an x-ray beam to excite nanophosphors to emit luminescent light, and three optical fiber bundles equiangular distributed upper surface of the phantom to collect the luminescence. The focused x-ray beam has a diameter of 50  μm, which was used for the simulation of x-ray imaging. The x-ray beam was translated with an increment of 0.5 mm in X-Y plane for 50 times to scan the phantom for each projection view. Total 15 projection views were acquired to generate sinogram for the tomographic imaging. Then the sinogram with 15 views was input to the trained ResNet model to synthesize new sinogram with 30 projection views. Then, the iterative image reconstruction algorithm with the minimization of the image total variation was applied to reconstruct nanophosphors concentration distribution from the synthesis sinogram. The nanophosphors distribution generates clearly patterns in sinogram, which is very effective to perform the interpolation task using deep learning techniques. Our results have shown that the proposed ResNet can effectively reduce noise and artifacts, and improve spatial resolution images, as shown in Fig. 3. The peak signal-to-noise ratio (PSNR) and SSIM are utilized to quantitatively evaluate the performance of the deep learning image processing methods, achieving PSNR of 24.09 and SSIM of 0.7993 for representative slices, whereas the image reconstructed with few-view projection data has PSNR of 19.48 and SSIM of 0.6968.

## 5 Conclusions and Future Works

XLCT has become an attractive imaging modality for the 3D high-spatial resolution imaging of deeply embedded x-ray excitable contrast agents with good measurement sensitivity. Here, we have first shown the design and build of our small-animal dedicated FXLT scanner that can perform both μCT imaging and 3D high-resolution XLCT imaging in one system. Having both the ability to perform the μCT imaging immediately before the XLCT imaging inside a single scanner is highly beneficial and allows for easier registration of the anatomical and optical imaging since the object orientations are guaranteed to be the same. With our previous setup, having to move the object to two different scanners for the imaging made it harder to ensure both CT and XLCT were scanned in the same orientations.^5^^–^^7^ A few other additions to the FXLT scanner will also allow for improved imaging performance compared with our prototype system in Ref. 7. First, the new scanner’s x-ray beam diameter is much smaller than the previous scanner (49.9 versus 101.5  μm), which will allow for higher spatial resolution capabilities. From our previous reports,^5^^,^^27^^,^^28^ we can estimate the spatial resolution of the designed FXLT scanner to be about 94  μm. Next, the use of four highly sensitive PMTs (H7422-50) to detect the emitted optical signals will allow for much more sensitive setup than previously where only one PMT was used. In addition, we have recently switched from silica core optical fiber bundles to liquid-light guides, which allow for higher optical transmission efficiency, especially in the NIR range, which means we expect much more signal to be delivered to each PMT than previously. From our previous study regarding the sensitivity of XLCT for ${\mathrm{Gd}}_{2}O_{2}S:{\mathrm{Eu}}^{3+}$, we estimated that the limits of detection (LOD) is approximately 2  μg/ml.^29^ With this new setup, we expect to achieve an even lower LOD, which will be verified in future studies. With additional PMTs, we are also capable of performing imaging of different nanoparticles with different emission wavelengths as well, which can be useful for co-registered imaging of multiple targets.

We have also synthesized 100 nm ${\mathrm{NaGdF}}_{4}$ (both Eu and Tb doped) nanophosphors with a silica coating and functionalized with biotin, which were successfully adhered to streptavidin-coated microspheres (Fig. 2). These particles have shown distinct luminescence spectra compared to previous ${\mathrm{Gd}}_{2}O_{2}S:{\mathrm{Eu}}^{3+}$ particles,^3^ which will allow for multi-color XLCT imaging. Lastly, we have also proposed and developed a deep-learning-based reconstruction algorithm for XLCT imaging based on the ResNet. In our preliminary work shown in this study, we can see a clear enhancement in the XLCT reconstruction image quality when comparing the few-view dataset image reconstruction [Fig. 3(d)] to the reconstructed full-view dataset [Fig. 3(f)] with our method.

Overall, in this work, we proposed, designed, and built the first reported small-animal dedicated FXLT scanner using a rotary gantry, synthesized ${\mathrm{NaGdF}}_{4}$ nanophosphors with unique emission spectra. We proposed a deep-learning-based XLCT reconstruction algorithm and have shown our preliminary results from these three aspects in this study. Our next direction with the FXLT scanner is to develop a hardware control and image acquisition software for performing both the μCT and FXLT scans. Future work with the nanophosphors involves optimizing synthesis, annealing, and surface modification protocols to obtain brighter nanophosphors and mixing with ${\mathrm{Gd}}_{2}O_{2}S:{\mathrm{Eu}}^{3+}$ nanophosphors for multicolor imaging. We will plan to first test and compare the already synthesized nanophosphors as potential XLCT imaging contrast agents by performing preliminary XLCT scanning using our previously developed system in Ref. 7. Later, we can develop nanospheres with brighter luminescence and longer stability, and their application will be tested as *in situ* light sources for detecting target receptor molecules *in vivo*. For the proposed deep-learning-based reconstruction algorithm, further testing will be performed to further verify our proposed reconstruction method particularly using datasets acquired from physical experiments. Finally, the ultimate goal will be to synergistically combine all aspects of this study (the FXLT scanner, synthesized nanophosphors, and developed reconstruction algorithm) by performing XLCT scanning of a mouse with our FXLT scanner, using developed nanophosphors as contrast agents, and then using our proposed algorithm to reconstruct the FXLT images.
